# EcoTILLING in *Capsicum *species: searching for new virus resistances

**DOI:** 10.1186/1471-2164-11-631

**Published:** 2010-11-12

**Authors:** Vicente P Ibiza, Joaquín Cañizares, Fernando Nuez

**Affiliations:** 1 Instituto de Conservación y Mejora de la Agrodiversidad Valenciana (COMAV), Universidad Politécnica de Valencia, 8E CPI, Camino de Vera s/n, 46022 Valencia, Spain

## Abstract

**Background:**

The EcoTILLING technique allows polymorphisms in target genes of natural populations to be quickly analysed or identified and facilitates the screening of genebank collections for desired traits. We have developed an EcoTILLING platform to exploit *Capsicum *genetic resources. A perfect example of the utility of this EcoTILLING platform is its application in searching for new virus-resistant alleles in *Capsicum *genus. Mutations in translation initiation factors (eIF4E, eIF(iso)4E, eIF4G and eIF(iso)4G) break the cycle of several RNA viruses without affecting the plant life cycle, which makes these genes potential targets to screen for resistant germplasm.

**Results:**

We developed and assayed a cDNA-based EcoTILLING platform with 233 cultivated accessions of the genus *Capsicum*. High variability in the coding sequences of the *eIF4E *and *eIF(iso)4E *genes was detected using the cDNA platform. After sequencing, 36 nucleotide changes were detected in the CDS of *eIF4E *and 26 in *eIF(iso)4E*. A total of 21 *eIF4E *haplotypes and 15 *eIF(iso)4E *haplotypes were identified. To evaluate the functional relevance of this variability, 31 possible eIF4E/eIF(iso)4E combinations were tested against *Potato virus Y*. The results showed that five new *eIF4E *variants (*pvr2^10^*, *pvr2^11^*, *pvr2^12^*, *pvr2^13 ^*and *pvr2^14^*) were related to PVY-resistance responses.

**Conclusions:**

EcoTILLING was optimised in different *Capsicum *species to detect allelic variants of target genes. This work is the first to use cDNA instead of genomic DNA in EcoTILLING. This approach avoids intronic sequence problems and reduces the number of reactions. A high level of polymorphism has been identified for initiation factors, showing the high genetic variability present in our collection and its potential use for other traits, such as genes related to biotic or abiotic stresses, quality or production. Moreover, the new *eIF4E *and *eIF(iso)4E *alleles are an excellent collection for searching for new resistance against other RNA viruses.

## Background

The *Capsicum *genus consists of 27 species [1], five of which have been domesticated and are used worldwide as vegetables, spices and condiments: *Capsicum annuum *L., *Capsicum frutescens *L., *Capsicum chinense *J., *Capsicum pubescens *R. & P. and *Capsicum baccatum *L. These species are cultivated around the world except for *C. pubescens*, which is only grown in the Andean Region. Nevertheless, non-pungent cultivars of *C. annuum *are the most-consumed, and are the main objective of most breeding programs [2]. *C. pubescens, C. baccatum, C. chinense *and *C. frutescens *are good sources of characters for the breeding of *C. annuum*, in addition to their economic and agronomic relevance. For instance, the *L *gene involved in resistance to *Tobacco mosaic virus *[3] was introgressed into modern *C. annuum *cultivars from *C. frutescens, C. chinense *and the wild species *Capsicum chacoense *H., while the *Tsw *gene, which determines local lesion response to *Tomato spotted wilt virus *[4], was introgressed from *C. chinense*. Moreover, resistance to *Cucumber mosaic virus*, identified in *C. baccatum*, has been employed to develop several tolerant cultivars in France and Hungary [5].

Genebanks maintain collections of different accessions that represent a great genetic wealth that can be explored for the breeding of traits or marker development. Nevertheless, the phenotypic characterisation of complete collections requires great effort. We need an alternative approach, one that is more efficient and less costly, in order to exploit this wealth in a practical way. EcoTILLING allows natural polymorphisms (Single Nucleotide Polymorphisms (SNPs) or small indels) to be identified quickly and easily in target genes [6]. The technique is based on the mutation detection approach used in TILLING (Targeting Induced Local Lesions in Genomes) [7,8]. The CEL I [9,10] or ENDO1 [11] endonucleases are used to recognise and cut all mismatched sites present in the heteroduplex of test and standard samples. In the last few years, several studies have used this high-throughput technology in different plants. Initially, Comai et al. adapted TILLING to a natural population of *Arabidopsis thaliana *H. [6] and named it EcoTILLING to denote that it tests natural variability and not mutagenised populations. The first study of a cultivated species was done on rice (*Oryza sativa *L.) [12]. EcoTILLING has been used in barley (*Hordeum vulgare *L.) [13], melon (*Cucumis melo *L.) [14], wheat (*Triticum aestivum *L.) [15], wild peanut (*Arachis duranensis *K. & G.) [16] and in the invasive aquatic plant *Monochoria vaginalis *B. [17]. EcoTILLING has also been used to detect SNPs in natural populations of several species [18-21].

As occurs with other vegetables, viral diseases are also the main limiting factor in pepper cultivation. The economic losses caused by viral diseases are quite significant, making the identification of new sources of viral resistance one of the main objectives of pepper breeders. Developing resistant cultivars provides an effective means for reducing the impact of diseases, but it requires constant effort as new virus isolates that overcome resistances are continuously produced in the host/parasite evolution race. Screening for new resistant sources in genebanks is a laborious and expensive task, and is a perfect example of the utility of EcoTILLING, as the technique allows accessions with different alleles of candidate genes to be identified and reduces the number of samples that need to be phenotyped.

The key to identifying new traits of interest using EcoTILLING is target gene selection. The cloning of several genes that confer recessive resistance to viruses has shown that they code for eukaryotic translation initiation factors of the 4F family. The factor 4F is made up of two subunits: eIF4E and eIF4G. An isoform of eIF4F, eIF(iso)4F, which is formed by the eIF(iso)4E and eIF(iso)4G subunits, has been identified in plants [22]. Alleles of *C. annuum *(*pvr2*), *C. chinense *(*pvr1*) and *Solanum habrochaites *S. & D. (*pot-1*) that confer resistance to *Potato virus Y *(PVY) and *Tobacco etch virus *(TEV) code for the translation initiation factor 4E, eIF4E [23-28]. Mutant alleles of the *eIF4E *gene block the multiplication of other members of the *Potyviridae *family in different crops: lettuce [29], pea [30,31], watermelon [32], barley [33] and common bean [34]. Another example of resistance to potyviruses are the EMS-induced mutant alleles at the *lsp1 *locus of *A. thaliana *that code for the eIF(iso)4E factor [35]. The combination of eIF4E and eIF(iso)4E mutations in pepper confers resistance to *Pepper veinal mottle virus *[36,37] and *Chilli veinal mottle virus *[38]. There are also examples of mutations in the eIF4E, eIF4G and eIF(iso)4G initiation factors that confer resistance to other viral families in different species: in melon (*Melon necrotic spot virus*, family *Tombusviridae*) [14], *A. thaliana *(*Cucumber mosaic virus*, family *Bromoviridae *and *Turnip crinkle virus*, family *Tombusviridae*) [39] and in rice (*Rice yellow mottle virus*, *Sobemovirus *Genus, family unassigned) [40]. All these data show that the interaction between plant translation initiation factors and viral RNA is necessary for viral replication and complete infection [41,42]. Studies have demonstrated that the interaction between eIF4E and VPg is necessary for potyviruses to complete their infectious cycle, and that eIF4E resistance-related factors alter the VPg binding function [24,25]. It is therefore apparent that certain mutations in translation initiation factors (eIF4E, eIF(iso)4E, eIF4G and eIF(iso)4G) break the virus cycle without affecting the plant life cycle. It is also clear that an interruption of viral infection can occur when a host factor is modified. For instance, mutations in the eIF4E factor inhibit the multiplication of *Potato virus Y *[23,25], one of the most aggressive pathogens of pepper worldwide. This virus causes distortion and mosaic on leaves and plant stunting, as well as deformation of fruits, producing severe yield reductions. Thus, these genes are potential targets in the search for new resistant sources to this and other viruses, and in identifying resistance alleles to effectively combat the appearance of new isolates that break resistance, as viral pathogens must generate a new function to overcome new resistance.

In this paper, we have developed a cDNA-based EcoTILLING platform in pepper. We have used cDNA to detect haplotype variants of the *eIF4E *and *eIF(iso)4E *genes inside CDS (coding sequence). This approach avoids intron sequences and reduces the difficulty of amplifying target genes from different species [14]. 36 and 26 nucleotide changes have been identified in *eIF4E *and *eIF(iso)4E*, respectively. Our results demonstrate that this platform contains enough variability to detect new mutations in the *eIF4E *and *eIF(iso)4E *genes in pepper. Moreover, to demonstrate the utility of these new allelic variants, we have tested them for PVY resistance. Five new resistance alleles of the *eIF4E *gene were detected. These results show the utility of this platform in searching for new alleles of other candidate genes, which will be very useful in pepper breeding.

## Results

### cDNA-based EcoTILLING platform

We analysed 229 cultivated accessions of the *Capsicum *genus (*C. annuum, C. pubescens, C*. *chinense, C. frutescens *and *C. baccatum*) and 4 accessions of two wild species (*C. chacoense *and *Capsicum eximium *H.) (see Additional file 1). These accessions were selected from the COMAV genebank according to their geographical origin and morphological variability. The accessions were collected from South America (Peru, Ecuador, Bolivia and Chile), where cultivated peppers show high morphological and genetic variability [43], and from the Canary Islands. We built up a cDNA platform in 96-well format.

### Screening of the *eIF4E *gene by EcoTILLING

The *eIF4E *gene in pepper is organised into five exons and shows a conserved exon/intron structure when compared to *A. thaliana *and rice [44]. We used the cDNA platform for the screening to detect *eIF4E *allelic variants. This strategy avoids the problem of intron localisation and variability and reduces the number of PCR and CEL I reactions. The completed ORF (open reading frame) was amplified by nested PCR, using two specific 3' UTR primers and a forward primer from the beginning of the CDS. A unique band was obtained in most of the accessions, but no amplification bands were obtained in 17 accessions of *C. pubescens*, 5 of *C. baccatum*, 4 of *C. frutescens*, 1 of *C*. *chinense *nor in any accessions of *C. eximium*, even though different amplification conditions were assayed. The amplified cDNA was quantified and used in the EcoTILLING reaction. In a first round of classification, all accessions were compared against the cDNA of the CDP04928 accession, and 36 different bands were detected. Samples were classified according to the presence or absence of these bands, including weak bands, in order to maximise the identification of very similar haplotypes. Finally, 55 different patterns were obtained and used to group the accessions. Sixteen samples showed no band and were classified with the CDP04928 group. The majority of groups were species-specific, and 34 groups contained only one accession. To confirm this initial classification, an internal standard sample was selected in the more numerous groups and common standards for the smaller groups with a similar band pattern. After doing the enzyme reactions with the new standards, a more detailed classification was done and the groups were reduced to 46.

To determine the allelic variants of the *eIF4E *gene, an accession from each group was selected and sequenced. After cDNA sequencing, the 46 groups were reduced to 29, as several EcoTILLING groups with similar band patterns and few samples were shown to have the same sequence. This discrepancy between EcoTILLING and sequencing groups could be explained by the presence of mutations in nearby sites, partial digestions, experimental errors and the inclusion of weak bands to maximise haplotype identification. In the end, 20 groups corresponded to homozygous samples and 9 to heterozygous samples. Most of the haplotypes were species-specific (see Table 1 and Additional file 1), but *eIF4E_B1 *was detected in *C. annuum *and *C. chinense*, *eIF4E_G1 *in *C*. *chinense *and *C. frutescens *and *eIF4E_F3 *was present in all three. The most variable species was *C. baccatum*, with eight alleles in both homozygous and heterozygous plants. In contrast, *C. pubescens *showed only one haplotype, *eIF4E_J1*. Seven heterozygous plants of *C. baccatum *were inferred from the haplotypes found in other accessions. In one instance of *eIF4E_Q1Sh1*, the *eIF4E_Sh1 *haplotype had not been detected in homozygosis. The other heterozygous plants were detected in *C*. *chinense *(*eIF4E_F3H1*) and *C. frutescens *(*eIF4E_F2G1*).

**Table 1:** Frequency of *eIF4E *haplotypes detected in *Capsicum *spp. by EcoTILLING.

**Haplotype**^a^	*C. annuum*	*C. chinense*	*C. frutescens*	*C. baccatum*	*C. pubescens*	*C. chacoense*
*eIF4E_A1*	13.3					
*eIF4E_B1*	46.7	2.5				
*eIF4E_C1*						100
*eIF4E_D1*		10				
*eIF4E_E1*	36.7					
*eIF4E_F1*				37.1		
*eIF4E_F2*			15.9			
*eIF4E_F2G1*			4.5			
*eIF4E_F3*	3.3	50	56.8			
*eIF4E_F3H1*		2.5				
*eIF4E_G1*		2.5	20.5			
*eIF4E_H1*		2.5				
*eIF4E_I1*		20				
*eIF4E_J1*					100	
*eIF4E_K1*				7.1		
*eIF4E_L1*				2.9		
*eIF4E_L1F1*				1.4		
*eIF4E_L1N1*				1.4		
*eIF4E_M1*				4.3		
*eIF4E_M1N1*				1.4		
*eIF4E_N1*				17.2		
*eIF4E_N1Q1*				1.4		
*eIF4E_O1*				8.6		
*eIF4E_O1F1*				5.8		
*eIF4E_P1*		10				
*eIF4E_Q1*				8.6		
*eIF4E_Q1F1*				1.4		
*eIF4E_R1*			2.3			
*eIF4E_Q1Sh1*^b^				1.4		

**a**, Haplotypes were named with a letter (coded protein) and a number (nucleotide variant). The heterozygous plants were named according to the two known haplotypes when identified in homozygosis. **b**, Heterozygote with one allele detected in homozygosis (*eIF4E_Q1*) and the other undetected (*eIF4E_Sh1*).

After the sequencing analysis, 34 polymorphic sites were detected in all exons except in exon 4. The number of changes per exon was 14 in exon 1, 5 in exon 2, 5 in exon 3 and 10 in exon 5. A total of 36 changes were identified, as two sites showed three different nucleotides. Nucleic acid sequences for each haplotype were translated, and 12 nucleotidic changes of the 36 were synonymous.

The sequences were aligned with the published alleles (data not shown). The sequenced ORFs from two haplotypes were identical to others previously found in different studies: the *eIF4E_E1 *nucleotide sequence was identical to the *pvr2^1 ^*allele of cultivar Yolo Y [Genbank: AF521964] [23], as was the *eIF4E_B1 *sequence to allele *pvr2^+ ^*carried by Yolo Wonder [Genbank: AY122052] [23] and Doux Long des Landes [Genbank: AF521963] [23].

### Analysis of the eIF4E amino acid sequence

The translation of nucleotide sequences resulted in 24 non-silent changes. However, these mutations resulted in 23 amino acid changes, as two mutations of *C. pubescens *affected the same codon (see Additional file 2). The 21 nucleotidic sequences from haplotypes were translated and 19 different predicted proteins were identified (Table 2). Most of the mutations were located in exon 1, where 12 amino acid substitutions were identified, five of which generated a change of charge in the amino acid. The majority of the changes were species-specific, but four mutations (N65 D, A73 D, L79R and T131I) were shared by different species (Table 1 and Table 2). *C. chinense *had the largest number of specific non-silent changes with six mutations (T51A, P66T, G107R, D213 H, L218R and L218F), whereas only one change was detected in *C. frutescens *(A15V) and *C. chacoense *(A15T).

**Table 2:** Differences in amino acid substitutions of detected haplotypes in the *eIF4E *gene.

eIF4E protein	New allele names	Published proteins		**Exon and amino acid position**^a^																			
		
		Allele name	Cultivar or accession	1	1	1	1	1	1	1	1	1	1	2	2	2	3	5	5	5	5	5	5
				15	51	65	66	67	71	73	74	77	79	107	109	131	160	213	214	216	218	219	221
eIF4E_B		*pvr2^+^*	Yolo Wonder	A	T	N	P	V	K	A	A	S	L	G	D	T	E	D	D	K	L	D	N
eIF4E_A		*pvr2^2^*	Dempsey	-	-	-	-	E	-	-	-	-	R	-	N	-	-	-	-	-	-	-	-
eIF4E_E		*pvr2^1^*	Yolo Y	-	-	-	-	E	-	-	-	-	R	-	-	-	-	-	-	-	-	-	-
eIF4E_R	*pvr2^17^*			V	-	-	-	-	R	-	-	-	-	-	-	-	-	-	-	-	-	-	-
eIF4E_C	*pvr2*^15^			T	-	-	-	-	R	-	-	-	-	-	-	-	-	-	-	-	-	-	-
eIF4E_Q	*pvr2*^22^			-	-	-	-	-	R	-	-	I	-	-	-	-	-	-	-	-	-	-	-
eIF4E_F		*pvr1^+^*	Habanero	-	-	-	-	-	R	-	-	-	-	-	-	-	-	-	-	-	-	-	-
eIF4E_G	*pvr2^16^*			-	-	-	-	-	R	-	-	-	-	-	-	I	-	-	-	-	-	-	-
eIF4E_L	*pvr2^19^*			-	-	-	-	-	R	-	-	-	R	-	-	-	-	-	-	-	-	-	-
eIF4E_J	*pvr2^18^*			-	-	-	-	-	R	-	-	-	-	-	-	-	G	-	E	A	-	E	-
eIF4E_N	*pvr2^13^*			-	-	D	-	-	R	-	D	-	-	-	-	-	-	-	-	-	-	-	-
eIF4E_O	*pvr2^14^*			-	-	D	-	-	R	-	-	-	-	-	-	-	-	-	-	-	-	-	-
eIF4E_H	*pvr2^11^*			-	-	D	-	-	R	-	-	-	-	-	-	-	-	H	-	-	R	-	-
eIF4E_K	*pvr2^12^*			-	-	D	-	-	R	D	-	-	-	-	-	-	-	-	-	-	-	-	-
eIF4E_Sh^b^	*-*			-	-	D	-	-	R	-	D	-	-	-	-	I	-	-	-	-	-	-	S
eIF4E_M	*pvr2^20^*			-	-	-	-	-	R	T	-	-	-	-	-	-	-	-	-	-	-	-	-
eIF4E_I		*pvr1*	PI152225	-	A	-	T	-	R	-	-	-	-	R	-	-	-	-	-	-	-	-	-
eIF4E_D	*pvr2^10^*			-	-	-	T	-	R	D	-	-	-	R	-	-	-	-	-	-	F	-	-
eIF4E_P	*pvr2^21^*			-	A	-	-	-	R	-	-	-	-	-	-	-	-	-	-	-	-	-	-

**a**, The alignment and amino acid positions are numbered according to the published eIF4E protein of *pvr2^+ ^*allele. **b**, Protein from *eIF4E_Sh1-*inferred haplotype was not named but had been used to analyse the variable positions.

The eIF4E proteins identified in our EcoTILLING platform as well as the proteins already published in the databases and articles were aligned with Clustal X (data not shown). Apart from the published haplotypes, *eIF4E_E1 *and *eIF4E_B1*, several partial proteins of the eIF4E factor were identical to already known proteins. Thus, the *eIF4E_F1*, *eIF4E_F2 *and *eIF4E_F3 *haplotypes with synonymous changes code for the same protein, eIF4E_F. This protein was the same as the eIF4E protein of cultivars that carry the *pvr1^+ ^*allele, Habanero [Genbank: AAR23917] [24] and Diego [Genbank: AAV88619]. The accessions with the eIF4E_A protein had the same amino acids as the *pvr2^2 ^*allele of cv. Dempsey [Genbank: AAR23920] [24], and the eIF4E_I protein was identical to the eIF4E protein of the *pvr1 *alleles identified in USDA accessions, PI159234 [Genbank: AAR23918] [24] and PI152225 [Genbank: AAV88618]. Finally, we detected 14 amino acid changes and 14 protein variants (including eIF4E_Sh) that had not been previously published (Table 2).

### Screening of the *eIF(iso)4E *gene by EcoTILLING

Amplification and analysis of the *eIF(iso)4E *coding sequence was carried out as with the *eIF4E *gene. The exon/intron structure of the *eIF(iso)4E *gene is unknown in pepper but has been described in tomato [Genbank: GQ451832, Genbank: GQ451833] and *A. thaliana *[Genbank: AF538308]. This initiation factor is organised into 5 exonic regions. The exon/intron structure will most likely be conserved in pepper, but the intron sequence most likely will not. As a result, to amplify the exons it will be necessary to sequence the intron boundaries in order to develop the eIF(iso)4E primers. The cDNA platform was therefore used to detect *eIF(iso)4E *allelic variants.

All samples were amplified and quantified by agarose gel. For this gene, the cDNA of the CDP06433 accession was chosen as reference and mixed with the other samples in the first grouping. After enzymatic digestion, a total of 21 bands were selected between 140 bp and 600 bp. All of these bands were used to classify the samples into 27 groups. To confirm the initial classification, new reactions were carried out using an internal standard for each large group and a common standard for related smaller ones. Finally, the samples were classified into 31 groups; of these, two groups contained 47% of the samples: one with 58% of the *C. baccatum *plants and another with 75% of the accessions of *C. chinense *and *C. frutescens*.

A sample from each group was selected and sequenced. After cDNA sequencing, the 31 groups were reduced to 17 because some EcoTILLING groups had the same sequence, as was previously detected for the *eIF4E *gen. These false positive rates were due to weak bands that were taken into account to minimise the haplotype loss. The *eIF(iso)4E *sequences of *Capsicum *showed a high level of polymorphism. Thus, 25 polymorphic nucleotide positions over the CDS were detected and 26 nucleotide changes were identified. One position had three different nucleotides.

A total of 15 haplotypes of the *eIF(iso)4E *gene were detected (see Table 3 and Additional file 1). All haplotypes were species-specific except *eIF(iso)4E_B1*, which was present in all accessions of *C. chinense *and was also the most common in *C. frutescens*. Only three heterozygous samples were detected, one in *C. annuum*, *eIF(iso)4E_D1C1*, and two in *C. pubescens*, *eIF(iso)4E_H1Ih1*, with inferred haplotype *eIF(iso)4E_Ih1 *not detected in homozygosis, and *eIF(iso)4E_Jhet*, with unknown phase. *C. pubescens *and *C. baccatum *were the most variable for the eIF(iso)4E initiation factor, as each showed between four and five different haplotypes. Only the *eIF(iso)4E_C1 *nucleotide sequence was identical to a previously published allele, *pvr6^+ ^*carried by FloridaVR2 [Genbank: DQ022082] [36], Yolo Y [Genbank: DQ022081] [36] and Yolo Wonder [Genbank: DQ022080] [36] cultivars. All the partial sequences of *eIF(iso)4E *ORFs (including *eIF(iso)4E_Jhet*) were translated, and 12 changes out of 26 were synonymous.

**Table 3:** Frequency of *eIF(iso)4E *haplotypes detected in *Capsicum *spp. by EcoTILLING.

**Haplotype**^a^	*C. annuum*	*C. chinense*	*C. frutescens*	*C. baccatum*	*C. pubescens*	*C. chacoense*	*C. eximium*
*eIF(iso)4E_A1*				25.3			
*eIF(iso)4E_B1*		100	79.2				50
*eIF(iso)4E_C1*	66.7						
*eIF(iso)4E_C2*			20.8				
*eIF(iso)4E_C3*				4			
*eIF(iso)4E_C4*				64			
*eIF(iso)4E_C5*				2.7			
*eIF(iso)4E_D1*	26.7						
*eIF(iso)4E_D1C1*	6.6						
*eIF(iso)4E_E1*						100	
*eIF(iso)4E_F1*				4			
*eIF(iso)4E_G1*					34.3		
*eIF(iso)4E_G2*					25.6		
*eIF(iso)4E_H1*					34.3		
*eIF(iso)4E_H1Ih1*^b^					2.9		
*eIF(iso)4E_Jhet*^c^					2.9		
*eIF(iso)4E_K1*							50

**a**, Haplotypes were named with a letter (coded protein) and a number (nucleotide variant). The heterozygous plants were named according to the two known haplotypes when identified in homozygosis. **b**, Heterozygote with one allele detected in homozygosis (*eIF(iso)4E_H1*) and the other undetected (*eIF(iso)4E_Ih1*). **c**, Heterozygote with unknown phase.

### Analysis of the eIF(iso)4E amino acid sequence

Fourteen non-synonymous changes were identified in homozygous and heterozygous individuals (Table 4). These mutations were located between the N-term of the protein and the middle region, up to the 119^th ^amino acid (see Additional file 3). Only three changes were shared by the different species: A20E (*C. pubescens *and *C. chacoense*), P27 S (*C. chinense *and *C. frutescens*) and A90T (*C. annuum*, *C. chinense *and *C. frutescens*); the other 11 mutations were specific to different species. Although only two accessions of *C. eximium *were sequenced, it was, along with *C*. *pubescens*, the species with the most specific mutations, as each had four changes. One non-synonymous change was detected in *C. chacoense *(T119I) as were two others in *C. baccatum *(P16T and A20T).

**Table 4:** Differences in amino acid substitutions of detected haplotypes in the *eIF(iso)4E *gene.

eIF(iso)4E protein	New allele Names	Published proteins		**Position of amino acid change**^a^										
		
		Allele name	Cultivar	15	16	20	24	27	56	63	90	109	115	119
eIF(iso)4E_C		*pvr6^+^*	F/YY/YW^b^	P	P	A	V	P	K	V	A	V	A	T
eIF(iso)4E_A	*pvr6^2^*			-	-	T	-	-	-	-	-	-	-	-
eIF(iso)4E_F	*pvr6^6^*			-	T	-	-	-	-	-	-	-	-	-
eIF(iso)4E_D	*pvr6^4^*			-	-	-	-	-	-	-	T	-	-	-
eIF(iso)4E_B	*pvr6^3^*			-	-	-	-	S	-	-	T	-	-	-
eIF(iso)4E_K	*pvr6^8^*			T	-	-	E	-	N	-	-	-	P	-
eIF(iso)4E_H	*pvr6^7^*			-	-	E	A	-	-	I	-	-	-	-
eIF(iso)4E_Ih^c^	*-*			-	-	E	S	-	-	I	-	-	-	-
eIF(iso)4E_G	*pvr6^9^*			-	-	E	A	-	-	-	-	-	-	-
eIF(iso)4E_E	*pvr6^5^*			-	-	E	-	-	-	-	-	-	-	I
eIF(iso)4E_Jh^c^	*-*			-	-	-/E	-/A	-	-	-/I	-	-/A	-	-

**a**, The alignment and amino acid positions are numbered according to the published eIF(iso)4E protein of *pvr6^+ ^*allele. **b**, F: Florida VR2.YY: Yolo Y. YW: Yolo Wonder. **c**. Proteins from inferred or unknown haplotypes were not named but were used to analyse the variable positions.

An alignment with the Clustal X program was carried out which included the partial eIF(iso)4E proteins detected and those already published. Besides the *eIF(iso)4E_C1 *sequence (*pvr6^+ ^*allele), the haplotypes *eIF(iso)4E_C2*, *eIF(iso)4E_C3*, *eIF(iso)4E_C4 *and *eIF(iso)4E_C5 *coded for the same protein, eIF(iso)4E_C, which was detected in 36% of the accessions. The other nine detected haplotypes did not correspond to any known protein variant.

### Response of selected accessions by EcoTILLING to mechanical inoculation with the F14K isolate of PVY

**Figure 1:**
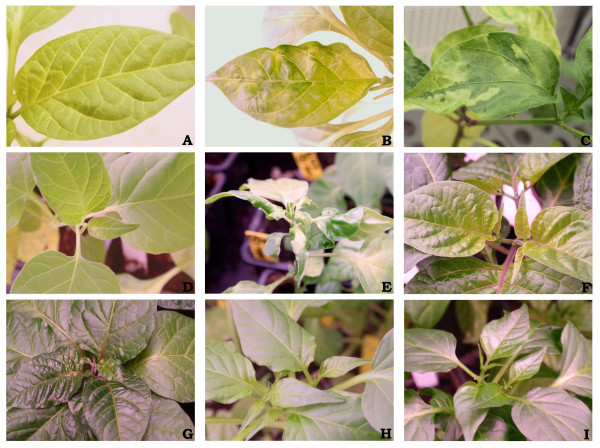
**PVY-F14K symptoms in pepper plants and representative resistant accessions**. (**A**) Agridulce negative control leaf at 15 DPI. (**B**) Agridulce leaf showing mosaic and deformation at 15 DPI. (**C**) Agridulce leaf showing dark green vein-banding between 45-60 DPI. (**D**) CDP08791 negative control (*C. chacoense*) at 45 DPI. (**E**) CDP08791 susceptible plant showing stunted growth at 45 DPI. (**F**) CDP02521 negative control (*C. chinense*) at 60 DPI. (**G**) CDP02521 resistant plant showing no symptoms at 60 DPI. (**H**) CDP09688 negative control (*C. annuum*) at 60 DPI. (**I**) CDP09688 resistant plant showing no symptoms at 60 DPI.

A screening with *Potato virus Y *was done to discover if the new *eIF4E *and *eIF(iso)4E *alleles detected in homozygosis confer resistance to this virus. In our EcoTILLING collection, we identified 18 eIF4E and 9 eIF(iso)4E proteins in homozygosis. We tested 33 accessions which represent 31 of the 32 eIF4E/eIF(iso)4E combinations (Table 5). These plants were inoculated mechanically with PVY-F14K isolate. The susceptible controls, Negral (CDP02198) and Agridulce (CDP05523), were infected and were DAS-ELISA positive, showing symptoms as of the beginning of the survey (15 days post inoculation (DPI)) (Table 5). These susceptible accessions showed leaf deformation, venal chlorosis and mosaic. Dark green vein-banding and stunted growth were detected later in these plants (Figure 1). These lesions were used to score the PVY symptoms from 0 for symptomless plants to 4 for dead plants. In Agridulce, the cultivar symptoms score and DAS-ELISA absorbance mean were higher than in Negral. Absorbance was higher than 0.60 in Agridulce plants and 0.32 in Negral. The F14K isolate of PVY completed the viral cycle in all analysed species and no plant died during the experiment (data not shown). The CDP01246, CDP04928 and CDP00620 accessions of *C. annuum*, CDP01263 and CDP01186 of *C. chinense*, CDP05838, CDP05436, CDP00973 of *C. frutescens *and the single accessions of *C. chacoense *(CDP08791) and *C. pubescens *(CDP00590) showed a similar response to that of the susceptible controls. The CDP00624 accession of *C. baccatum *showed a susceptible response too, but the symptoms were milder than in other susceptible plants, showing a maximum symptom score of 0.5. The susceptible accessions showed a maximum symptom index between 0.5 and 3.5 and a mean maximum absorbance between 0.61 and 1.81. CDP00614, CDP05581, CDP07825 and CDP04710 accessions of *C. baccatum *and CDP06951 of *C. frutescens *showed 100% systemically infected plants with absorbances higher than the threshold of each accession (A_405 _= 0.068-0.089) (date not shown), but no symptoms of systemic infection were detected throughout the survey. These accessions were considered tolerant to PVY-F14K. Moreover, CDP06360 of *C. baccatum *and CDP07700 of *C*. *chinense*, which showed 60% plants with systemic infection without symptoms, were also considered tolerant. Four accessions of *C. annuum *(CDP06188, CDP09688, CDP06433 and CDP01135), 2 of *C. chinense *(CDP02521 and CDP08407) and 9 of *C. baccatum *(CDP06234, CDP04291, CDP02320, CDP04865, CDP07490, CDP04131, CDP09967, CDP09334 and CDP05929) showed the best response; no plants were infected systemically (all showed a DAS-ELISA negative) and no symptoms were detected (Figure 1). These accessions were considered resistant to the F14K isolate of *Potato Virus Y*.

**Table 5:** Development of PVY-F14K infection during screening and response in selected accessions.

Accession	Species	eIF4E/eIF(iso)4E proteins	**Mean symptom index**^a^				**Mean absorbance (A_405_)**^a^				**% positive plants**^c^	**Response**^d^
					
			**15 D**^b^	30 D	45 D	60 D	15 D	30 D	45 D	60 D		
CDP06188	*C. annuum*	AC	0	0	0	0	0.05	0.03	0.03	0.02	0	R
CDP09688	*C. annuum*	B_CD	0	0	0	0	0.02	0.02	0.02	0.02	0	R
CDP01263	*C. chinense*	BB	3.5	2.5	1.5	1	1.24	0.89	0.65	0.29	100	S
CDP04928	*C. annuum*	BC	0	0	0.5	1	0.07	0.46	0.72	0.37	100	S
CDP01246	*C. annuum*	BD	0.5	0.9	1.1	1.5	0.27	0.51	0.61	0.57	100	S
CDP08791	*C. chacoense*	CE	2	2.5	3.5	3.5	0.85	1.81	1.73	1.79	100	S
CDP02521	*C. chinense*	DB	0	0	0	0	0.04	0.03	0.02	0.02	0	R
CDP06433	*C. annuum*	EC	0	0	0	0	0.03	0.02	0.02	0.05	0	R
CDP01135	*C. annuum*	ED	0	0	0	0	0.03	0.02	0.01	0.01	0	R
CDP00614	*C. baccatum*	FA	0	0	0	0	0.54	0.25	0.17	0.26	100	T
CDP05838	*C. frutescens*	FB	0	1.6	1	0.5	0.76	0.67	0.52	0.97	100	S
CDP00624	*C. baccatum*	FC	0	0.5	0.5	0	0.87	0.35	0.14	0.35	100	S
CDP06234	*C. baccatum*	FC	0	0	0	0	0.04	0.03	0.02	0.03	0	R
CDP00620	*C. annuum*	FD	0.9	1.9	2.3	1.9	0.64	1.01	1.37	1.12	100	S
CDP05436	*C. frutescens*	GB	0	0.9	1.3	1	1.36	0.88	0.98	1.33	100	S
CDP00973	*C. frutescens*	GC	0	0.8	1	0.8	1.21	0.66	1.39	1.09	100	S
CDP08407	*C. chinense*	HB	0	0	0	0	0.01	0.01	0.01	0.03	0	R
CDP07700	*C. chinense*	IB	0	0	0	0	0.26	0.22	0.03	0.03	60	T
CDP00590	*C. pubescens*	JG	2	2.4	1	0.2	1.81	1.6	0.68	1.55	100	S
CDP04291	*C. baccatum*	KA	0	0	0	0	0.03	0.04	0.03	0.02	0	R
CDP02320	*C. baccatum*	KC	0	0	0	0	0.03	0.03	0.02	0.04	0	R
CDP05581	*C. baccatum*	LC	0	0	0	0	1.64	1.13	0.76	0.32	100	T
CDP06360	*C. baccatum*	MC	0	0	0	0	0.07	0.08	0.09	0.09	60	T
CDP07825	*C. baccatum*	MF	0	0	0	0	0.43	0.41	1.61	1.32	100	T
CDP04865	*C. baccatum*	NA	0	0	0	0	0.03	0.04	0.04	0.06	0	R
CDP07490	*C. baccatum*	NC	0	0	0	0	0.05	0.02	0.02	0.03	0	R
CDP04131	*C. baccatum*	NF	0	0	0	0	0.05	0.05	0.04	0.06	0	R
CDP09967	*C. baccatum*	OA	0	0	0	0	0.04	0.04	0.02	0.02	0	R
CDP09334	*C. baccatum*	OC	0	0	0	0	0.03	0.04	0.03	0.04	0	R
CDP01186	*C. chinense*	PB	2.5	3	3	3.5	0.88	0.19	0.23	0.25	100	S
CDP05929	*C. baccatum*	QA	0	0	0	0	0.03	0.02	0.02	0.01	0	R
CDP04710	*C. baccatum*	QC	0	0	0	0	0.33	0.18	0.13	0.16	100	T
CDP06951	*C. frutescens*	RB	0	0	0	0	1.44	0.77	1.21	1.34	100	T
CDP02198	*C. annuum*	Control	0.7	1.7	1.5	1.9	0.41	0.34	0.33	0.32	100	S
CDP05523	*C. annuum*	Control	2.1	2.8	3.2	3.5	0.88	0.6	0.7	1.27	100	S

**a**, Arithmetic mean calculated from all plants of the accession at each time sampling. The symptom index ranged from 0 (symptomless plant) to 4 (dead plant). **b**, D: Days post inoculation. **c**, Percentage of DAS-ELISA positive plants. Plants were considered to be DAS-ELISA positive when absorbance (405 nm) of sample from the youngest leaf was higher than three times the negative control mean, and classified as non-infected (DAS-ELISA negative) when the value of absorbance was below this threshold. The negative control mean was calculated for each accession with different time samplings (15, 30, 45 and 60 days post inoculation). **d**, R: Resistant. No plants were infected systemically and no symptoms were identified. S: Susceptible. Showed DAS-ELISA positive and systemic symptoms. T: Tolerant. Showed DAS-ELISA positive in all or some plants but without symptoms.

## Discussion

### High polymorphism in pepper EcoTILLING platform

We developed a cDNA EcoTILLING platform for pepper to search for allelic variants of the *eIF4E *and *eIF(iso)4E *genes. 233 accessions from South America and the Canary Islands were selected in their primary (*C. chinense*, *C. frutescens, C. pubescens *and *C. baccatum*) or secondary (*C. annuum*) centres of diversity [5]. Maximum variability is found within these centres of diversity, which has made it possible to find a high polymorphism in the eIF4E and eIF(iso)4E initiation factors.

36 nucleotide changes were detected in the 21 coding sequences of the *eIF4E *gene as well as 26 changes in the 17 *eIF(iso)4E *sequences. Our results showed that the pepper accessions used are highly variable. It is possible that cultivated peppers have more genetic variability compared to other crops, such as tomato [45]; but it is also possible that this high variability is due to our selection, based as it is on the diversity centres as opposed to a worldwide selection. The only other EcoTILLING study with the *eIF4E *gene done in melon found very low diversity [14]. Only six polymorphic sites were identified in 113 accessions of *C. melo *and one accession of *Cucumis africanus *L. In spite of the fact that our EcoTILLING assays were only done in exons, our pepper cDNA platform has a high level of polymorphism compared to melon, and is similar to other works that use EcoTILLING in wild or natural species with other genes. Comai et al. used 150 plants of *A*. *thaliana *and discovered 55 haplotypes in the five loci analysed [6]. In 25 natural variants of *Mla *in barley, from five to ten point mutations in 451 bp were identified [13]. EcoTILLING has also identified polymorphisms in black cottonwood (*Populus trichocarpa *T.), and 63 SNPs were identified in 8191 bp [18]. In *Vigna radiata *L., a total of 131 SNPs and 26 indels in 45 haplotypes from ten primer sets (6461 bp) were detected [19]. In common bean (*Phaseolus vulgaris *L.), 22 SNPs were identified in 37 EST candidates [21], whereas in 30 accessions of peanut, eight SNPs were identified in an amplicon of 1280 bp [17]. Therefore, our EcoTILLING collection has high genetic variation between selected accessions, which could be very useful for identifying natural variation in other genes related to biotic or abiotic responses and quality.

### Variability of *eIF4E *and *eIF(iso)4E *genes in pepper

A total of 21 haplotypes of the *eIF4E *gene and 15 haplotypes of the *eIF(iso)4E *gene were identified. Although most of the haplotypes were species-specific, *eIF4E_B1 *was detected in *C*. *annuum *and *C. chinense*, *eIF4E_G1 *in *C. chinense *and *C. frutescens, eIF4E_F3 *was present in all three of these species and *eIF(iso)4E_B1 *was present in *C. chinense, C. frutescens *and *C. eximium*. This may be due to the genetic flow between the species of the *C. annuum *complex (*C. annuum*, *C*. *chinense *and *C. frutescens*) [46]. However, in the case of *C. eximium*, it is likely due to the presence of ancestral haplotypes, as this species does not cross viably with the others [47].

The 21 *eIF4E *nucleotide sequences coded for 19 different proteins, and the 15 *eIF(iso)4E *haplotypes coded for 10 eIF(iso)4E proteins, 23 of which were new protein variants. In previous works, 10 allelic variants of eIF4E proteins in *C. annuum *[23,25] and 2 allelic variants in *C*. *chinense *[24] that coded for 12 eIF4E proteins were described. The published proteins of *C. chinense *and three of *C. annuum *were also identified by EcoTILLING in our pepper collection. In the case of the eIF(iso)4E initiation factor, only 2 proteins in *C. annuum *had been previously described [36]; our *eIF(iso)4E_C1 *haplotype was identical to the published allele, *pvr6*^+^. We identified 14 new eIF4E proteins and 9 new eIF(iso)4E proteins in our pepper collection, but alleles and proteins that had already been described were also identified, confirming that our EcoTILLING platform contains a good representation of *Capsicum *variability.

We detected 24 non-synonymous changes in *eIF4E *haplotypes and 14 in *eIF(iso)4E*. This high number of amino acid substitutions might be due to the selection and co-evolution of virus and host. In fact, Charron et al. described the evidence for co-evolution between eIF4E of *C. annuum *plants and potyviral VPg, as there is a strong evolutionary pressure to resist viral pathogens [25]. Amino acid changes in the central domain of the PVY-VPg protein have been demonstrated to be subject to positive selection [48]. Moreover, the existence of positive selection within the recessive resistance gene *eIF4E *has already been described in plants [49].

### Functionality of new alleles identified by EcoTILLING

The main objective of EcoTILLING is to isolate useful new haplotypes or alleles in target genes. Thus, the molecular variation in *eIF4E *and *eIF(iso)4E *genes could be very useful for identifying new resistance alleles against important viruses in pepper. Among the pepper potyviruses, *Potato virus Y *is widespread throughout most of the cultivated areas [25]. PVY can be transmitted by many species of aphids, but chemical methods are effective enough to control the vector. Nevertheless, the impact of this virus has increased due to the restriction of phytosanitary treatments. In recent years, resistance alleles for this virus have not been very important in breeding programs, but now, with the treatment reduction, the use of resistant varieties is the most effective way to prevent damage from the virus. Therefore, a screening with PVY-F14K was done to study the response of the new eIF4E and eIF(iso)4E proteins discovered. The F14K isolate of PVY completed the viral cycle in some or all accessions of each analysed species, which indicated that resistance is not species-specific.

We analysed the results according to the protein combinations of eIF4E and eIF(iso)4E (see Additional file 4). From analysis of the correlation between proteins and disease resistance, we hypothesised that the eIF(iso)4E proteins were not involved in the resistance to PVY as most of the accession responses could be explained by eIF4E proteins. Accessions carrying the proteins eIF4E_C, eIF4E_F, eIF4E_G, eIF4E_J and eIF4E_P generated symptoms, while accessions with the proteins eIF4E_M, eIF4E_L, eIF4E_R and one accession with the eIF4E_Q protein (CDP04710) and another with eIF4E_F (CDP00614) showed systemic infection but were symptomless. Other accessions carrying new eIF4E proteins found in this study (eIF4E_D, eIF4E_H, eIF4E_K, eIF4E_O and eIF4E_N) showed a resistance response as the viral infection was not detected throughout the experiment.

When we compared previous results of the published eIF4E proteins, we obtained the expected response in some accessions that contain these eIF4E proteins. Thus, the accessions with eIF4E_E and eIF4E_A proteins did not show systematic infection, like the *pvr2^1 ^*and *pvr2^2 ^*alleles that break the PVY cycle when inoculated [23,25,48]. The eIF4E_I protein was identical to the eIF4E protein that carries the *pvr1 *allele that confers broad-spectrum resistance to strains of PVY [50]. In spite of this, the CDP07700 accession (eIF4E_I protein) showed a tolerant response, as 3/5 of the plants were DAS-ELISA positive without symptoms. Nevertheless, all CDP07700-accession plants blocked viral accumulation at 45 and 60 DPI. Different factors may explain the initial viral accumulation in some plants of this accession, such as environmental or experimental factors (heavy inoculation pressure, development stage, temperature) or distinct responses to different PVY isolates. All accessions that contain the eIF4E_B protein, identical to the *pvr2^+ ^*susceptible allele [23,25], showed a susceptible response to PVY-F14K, except the CDP09688 accession that did not show PVY systemic infection and in which no symptoms were observed. The resistance response of this accession suggests that it is not related to eIF4E nor to eIF(iso)4E, as accessions that carry the same proteins in homozygosis (eIF4E_B, eIF(iso)4E_C or eIF(iso)4E_D) showed susceptible, tolerant or resistant responses. Thus, this response to PVY could be due to another resistance mechanism.

The CDP06234 accession with the eIF4E_F and eIF(iso)4E_C proteins did not show viral multiplication in all tested plants, unlike the other accession with the same protein variants of both genes, CDP00624. The resistance response of the CDP06234 *C. baccatum *accession could not be explained by these translation initiation factors. The CDP00614 accession that carries the eIF4E_F and eIF(iso)4E_A proteins showed a different response compared to other accessions with the same eIF4E protein variant. These plants showed systemic infection but were symptomless. Nevertheless, in this case, the combination of eIF4E_F and eIF(iso)4E_A could not be ruled out as no accessions with eIF(iso)4E_A showed a susceptible response. Moreover, eIF(iso)4E_A could also explain the different responses of the accessions with the eIF4E_Q protein, CDP05929 and CDP04710. Nevertheless, this combination hypothesis is unlikely as the eIF(iso)4E factor was not related to resistance to PVY in the previous works [23,25] and could only clarify the response of these two latter accessions. In fact, the response of CDP09688, CDP06234, CDP00614 and CDP05929 to PVY-F14K is more easily explained by the presence of another gene or resistant mechanism. For instance, the *Pvr4 *anonymous locus of the *C. annuum *line, Criollo de Morelos 334, showed a complete dominant resistance to all PVY pathotypes as well as to *Pepper mottle virus *[51].

Nine newly identified eIF4E proteins seemed to interact with PVY-F14K, but five new eIF4E variants (D, H, K, N and O) were related to resistance response to this virus. The published resistance alleles of pepper carry different combinations of non-conservative amino acid changes that are localised on the surface of *eIF4E *in regions I (exon 1) and II (exon2) [25,41]. The changes in these regions seem to be responsible for resistance at least against PVY [23,25]. Thirteen non-synonymous changes (amino acid positions 15, 65, 73, 77, 131, 160, 213, 214, 216, 218 and 219) of the *eIF4E *haplotypes analysed by mechanical inoculation in this survey were previously unknown (Table 2). Seven of the mutations detected by EcoTILLING in exons 1 and 2 of the *eIF4E *gene generate a change of charge. Six mutations are specific to the new resistance alleles and are localised in exon 1 or exon 5. The published proteins eIF4E_A (*pvr2^2^*) and eIF4E_E (*pvr2^1^*) have a V67E change (region 1), which is sufficient to compromise PVY infection [25]. eIF4E_N, eIF4E_O and eIF4E_K of *C. baccatum *and eIF4E_ H of *C. chinense *have a single amino acid substitution in exon 1 (N65D) which also may prevent multiplication of the virus. Although *C. baccatum *showed a low susceptibility to PVY-F14K, this mutation may explain the resistance response of some *C*. *baccatum *accessions. The other resistant protein, eIF4E_D (*C. chinense*), has other amino acid changes located in regions I (P66T, A73D), II (G107R) and V (L218F). These changes in regions I and II are also present in other resistance or tolerance eIF4E proteins (eIF4E_K and eIF4E_I).

The new alleles of the eIF4E initiation factor have been named *pvr2 *with a numerical superscript for each new allele based on the latest described allele (*pvr2^9 ^*of Chile de Arbol (*C. annuum*) [25]), using the nomenclature of Kyle and Palloix [50]. The resistance alleles to PVY-F14K, *D1*, *H1*, *K1*, *N1 *and *O1*, have been designated as *pvr2^10^*, *pvr2^11^*, *pvr2^12^*, *pvr2^13 ^*and *pvr2^14^*, respectively. The new susceptible or tolerant alleles of the *eIF4E *gene, *C1*, *G1*, *R1*, *J1*, *L1*, *M1*, *P1 *and *Q1*, have been designated as *pvr2^15^*, *pvr2^16^*, *pvr2^17^*, *pvr2^18^*, *pvr2^19^*, *pvr2^20^*, *pvr2^21 ^*and *pvr2^22^*, respectively. The new *eIF(iso)4E *alleles have been named *pvr6 *with a numerical superscript for the new allele based on the only resistance allele, *pvr6 *(Perennial [Genbank: DQ022083] cultivar of *C. annuum *[36]). Thus, the *eIF(iso)4E *alleles that code for a new eIF(iso)4E protein, *A1*, *B1*, *D1*, *E1*, *F1*, *H1 *and *K1*, have been designated as *pvr6^2^*, *pvr6^3^*, *pvr6^4^*, *pvr6^5^*, *pvr6^6^*, *pvr6^7 ^*and *pvr6^8^*. Finally, the alleles that code for the eIF(iso)4E_G protein (*eIF(iso)4E_G1 *and *eIF(iso)4E_G2*) have been named *pvr6^9^*.

These five new resistant *eIF4E *alleles, and their possible use in heterozygosis, could make their introgression into new commercial hybrids easier. Moreover, these recessive alleles are excellent allele reserves against the changing nature of viral pathogens, as mutations are required in the *VPg *gene of the PVY to restore its interaction with the different mutated eIF4E proteins [25,48].

### EcoTILLING platform implemented in pepper

The use of cDNA as starting material for endonuclease-based EcoTILLING has not been previously described. However, the cDNA-based mutation screening has been described using other technologies, such as HRM [52] or DNA sequencing [53]. This strategy has several advantages with respect to EcoTILLING assays based on DNA. Although EcoTILLING is a potent technique for discovering SNPs and examining DNA variation in natural populations, one problem is the amplification of target genes from wild species, due to sequence mispriming [14]. The use of cDNA and primers located in transcribed sequences avoids this problem, as these regions are more conserved. This facilitates the use of sequence data from related and model species. Another advantage is that this system avoids the intron sequence, and the complete CDS can be amplified in one or two reactions. In DNA EcoTILLING, the presence of introns may be a problem as most of the changes will be located in these regions. To avoid this, an exon-by-exon strategy is usually adopted, requiring as many reactions as there are exons in the candidate gene. In contrast, the cDNA EcoTILLING approach reduces the number of PCR and CEL I reactions per sample.

This work is an example of where cDNA EcoTILLING is both cost- and time-effective: it involves different species, only mutations in the CDS are of interest and the genes have several highly variable exons. Another question is the possibility of using oligo-dT in order to transcribe several genes with the same RT reaction. We used specific primers to improve the efficiency and specificity of the amplification of candidate genes. The expression of the candidate genes in the target tissue has to be previously studied.

One of the essential points of CEL I-based EcoTILLING is the analysis of the band pattern and classification of the samples in relation to it. Some factors may make pattern identification in EcoTILLING assays more difficult. One is experimental variation, as different reactions and denaturing gels make band comparison more difficult. Another factor is the differential mismatch preference shown by the endonuclease, CEL I [9], which may also be influenced by experimental variance, resulting in different band intensity in different samples. It is of note that this preference was not observed by Till et al. using IRDye labelling and the LI-COR system [10]. Some factors depend on target sequence, for if there are several mismatches between the samples and the standard sample, the detection of all internal cuts is difficult because the cDNA is only labelled at the 5' and 3' terms. Thus, the presence of several internal cut sites produces weak bands due to partial digestion. Moreover, very close mismatches are very difficult to differentiate by electrophoresis, especially if, due to the number of samples involved, several gels are used. The use of a second standard with a similar or identical sequence reduces these problems and facilitates the correct classification of the samples. These problems, related to accuracy, are not associated with complete CDS amplification; even if the analysis is carried out independently for each exon, it is necessary to use a second standard to detect the presence of very close mutations.

Thus, to confirm our initial classification, the samples were compared to a new related standard. After the second enzyme reaction, the samples were reclassified and the groups reduced to 46 for the *eIF4E *gene and increased to 31 for *eIF(iso)4E*. Nevertheless, after cDNA sequencing, the groups were reduced to 29 and 17, respectively. This discrepancy between CEL I groups and sequencing haplotypes is attributable to small differences in band pattern due to experimental variation, the high variability of *eIF4E *and *eIF(iso)4E *genes in the selected accessions and the presence of very close mutations. To minimise rare allele loss we designed our double-test protocol and took into account a large number of weak bands, which increased the false positive rate. This explains why most CEL I groups were represented by one or two accessions and why some of them showed the same sequence. This would also happen if a genomic DNA platform were used. In spite of the number of false positives in our assay, this double test is very efficient in detecting differences between very similar haplotypes. In our analysis, we detected six haplotypes that had been misclassified in the first analysis, one of which resulted in an *eIF4E *allele resistant to PVY-F14K.

This EcoTILLING platform contains a good representation of *Capsicum *genetic variability and is easy and efficient to test using CEL I-based protocols. But this collection is also useful in identifying new nucleotide variations using other techniques, such as direct sequencing with new sequencing systems (NGS), high resolution melting (HRM) or sensitive capillary electrophoresis (CSCE). Several new approaches based on these techniques have been published [54-56], and it is very likely that more efficient NGS-based strategies will be developed. For this collection, we have also built up a DNA platform in 96-well format available for use. In this new scenario, our pepper platforms with cDNA and DNA will facilitate and still be useful for the isolation of new mutations and SNPs related to traits useful in the breeding of these species.

## Conclusions

EcoTILLING has been optimised in different species of pepper and is the first to use cDNA and not genomic DNA; this approach avoids intronic sequence problems and reduces the reaction number. We analysed 233 accessions looking for allelic variants of the *eIF4E *and *eIF(iso)4E *genes. High variability for these initiation factors was detected, which showed a high genetic variation between selected accessions when screened by EcoTILLING. Therefore, genes related to biotic or abiotic responses and quality (pungency level, extractable red colour and flavour) are potential targets using our cDNA or DNA platforms. Five new resistance alleles were identified for PVY, and could be introgressed into commercial pepper cultivars to contribute to a more effective and stable resistance to different PVY isolates. This *eIF4E*/*eIF(iso)4E *haplotype selection is also a source for searching for new resistance alleles in the fight against other viruses.

## Methods

### Plant material screening by EcoTILLING

The accessions of *Capsicum *genus included 30 accessions of *C. annuum*, 41 of *C. chinense*, 48 of *C. frutescens*, 75 of *C. baccatum*, 35 of *C. pubescens*, 2 of *C. eximium *and 2 of *C. chacoense *(see Additional file 1). These accessions were obtained from the COMAV genebank and were collected from Bolivia, Ecuador, Chile, Peru and the Canary Islands (Spain). The accessions were selected according to their morphological variability and geographic origin. All seeds were treated with 10% (w/v) Na_3_PO_4_·12H_2_O during 3 hours to prevent viral infection, and with 3% (w/v) sodium hypochlorite during 10 min to sterilise and facilitate germination. A germinated seed from each accession was transferred to a 7 × 7 × 6.5 cm polyethylene pot and grown under greenhouse conditions.

### RNA isolation

RNA extraction from 75 mg of young pepper leaves was carried out with TRI-Reagent protocol (Sigma-Aldrich, Saint Louis, Missouri, USA). Total RNA was determined spectrophotometrically using a Biophotometer (Eppendorf, Hamburgo, Germany) and was stored at -80°C. Reverse transcription was carried out with these RNA samples to build up a cDNA-based EcoTILLING platform in 96-well format.

### Reverse transcription and amplification

Specific primers for the amplification of full-length *eIF4E *ORF were designed according to the published sequence [GenBank: AY122052] (*C. annuum*); for *eIF(iso)4E *ORF we used TC5256-EST (TIGR CaGI) (*C. annuum*). First-strand cDNA was synthesised using Expand Reverse Transcriptase (Roche, Mannheim, Germany) according to the manufacturer's protocol. RT was performed at 43°C for 60 min using 1 μg total RNA and 50 pmol of 3' UTR specific primers, GTGCCTACAACTTTTCAGTACG for *eIF4E *gene and CAAGTATTGCTGGAACTTGG for *eIF(iso)4E*. These were used to improve the efficiency and specificity of the amplification of candidate genes. The completed open reading frames were amplified by nested PCR. The PCR was performed in a final volume of 50 μl from 2.5 μl of RT reaction, using DNA polymerase Netzyme (N.E.E.D, Valencia, Spain) and primers from the beginning of the coding part of the *eIF4E *(ATGGCAACAGCTGAAATGG) and *eIF(iso)4E *genes (ATGGCCACCGAAGCACCAC). The reverse primers were those used in the RT reaction. PCR cycling conditions were 94°C 5 min, (94°C 1 min, 58°C 30 sec, 72°C 1 min) for 15 cycles and 72°C 10 min. The second PCR was carried out using the same forward primers, but the reverse primers were CTTATATTCCGACATTGCATC (*eIF4E *gene) and GAGGGGTCAATCCCACACG (*eIF(iso)4E *gene). The second PCR was carried out from 2.5 μl of each first PCR, using the same conditions as the first PCR but repeated 25 cycles. PCR product was checked by 1% agarose gel electrophoresis and DNA concentration was estimated by densitometry.

### Screening for polymorphisms and sequence

The celery juice extraction was carried out as described by Till et al. [10]. EcoTILLING reactions were carried out according to the procedure described by Comai et al. [6], with certain modifications. In contrast to standard EcoTILLING procedure, each PCR reaction was performed independently to avoid problems related to differences in the amplification efficiency of target genes from two species in the same reaction. Thus, 100 ng of PCR product from each sample and 100 ng of labelled standard sample were mixed in a final volume of 15 μl using 96 well plates. Standard samples were amplified just like normal samples, but using both IRDye-700-labelled primers in the nested PCR. These samples were selected as they belonged to the most common group of accessions that were previously tested. DNA duplexes were obtained by heat-denaturing at 95°C for 10 min and cooled slowly to 25°C with a rate of 0.1°C/s. These duplexes were digested with 5 μl of a diluted (1:5) celery extract during 20 min at 42°C. EcoTILLING reactions were stopped by adding 2.5 μl 0.5 M EDTA, purified by precipitation with absolute ethanol and 5 M NaCl and resuspended in 10 μl of formamide with bromophenol blue. Reactions were resolved using a LI-COR 4300 DNA Analyzer (Biosciences, Lincoln, Nebraska, USA) with 48 well comb and 0.25 mm 6% acrylamide denaturing gels. The gels were run at 1500 V, 40 W, 40 mA, 45°C for 3 h for the *eIF(iso)4E *gene and 3.5 h for *eIF4E*. Analysis of the gel images was carried out manually using GIMP 2.4.7 (Free Software Foundation, Boston, Massachusetts, USA). A binary matrix was generated reflecting the presence (1) or absence (0) of each band for each sample. Accessions with no cut bands were assigned to the same group as the standard sample, and those that showed the same band patterns were grouped together. To confirm the first classification, all accessions were compared to a new standard but it was not necessary to perform another PCR for test samples. Thus, an internal standard sample was selected from each large group. However, groups with few samples and similar patterns were reclassified using the same standard. Two LI-COR gel images of the first and second classification are shown (see Additional file 5 and 6). The *eIF4E *and *eIF(iso)4E *genes from one accession of each final group were sequenced from both ends with an ABI PRISM 3100 Avant Genetic Analyzer (Applied Biosystems, Woolston, UK). The sequences were cleaned and assembled using the Staden software package http://staden.sourceforge.net/staden_home.html. Nucleotide and amino acid sequences were analysed with the EMBOSS package http://emboss.sourceforge.net/. The Clustal X (1.83) algorithm [57] was used to multiple sequence alignments. Haplotypes of heterozygous plants were inferred from different haplotypes sequenced in homozygosis. All sequences were deposited in the EMBL nucleotide database with reference numbers from FN824321 to FN824348 and FN813352 for the *eIF4E *gene and from FN824349 to FN824364 and FN813353 for the *eIF(iso)4E *gene. A plant from each haplotype detected in homozygosis was grown under greenhouse conditions and seeds were obtained by self-pollination.

### PVY inoculation procedure

From each selected haplotype, 4 or 5 plants were tested against the isolate F14K of PVY, kindly provided by Dr. J. Aramburu (IRTA, Cabrils, Spain). Two cultivars of *C. annuum *(Negral and Agridulce) were used as susceptible controls to PVY. Six days after germination, plants were transferred to 9 × 9 × 8 cm pots in a growth room with controlled environmental conditions: 25/18°C (day/night) temperature, 60/85% (day/night) relative humidity, 60-85 μmol s^-1 ^m^-2 ^of irradiance from Sylvania Gro Lux fluorescent tubes and a photoperiod of 14/10 hours (light/darkness).

The inoculum was prepared from reservoir line leaves, Fortuna C of *Solanum lycopersicum *L. PVY isolate was maintained on this line and transferred to young plants every 4-5 weeks. 2 g of tissue were ground in 20 ml of inoculation buffer (0.15 M NaCl, 3 mM NaH_2_PO_4_, 75 mM Na_2_HPO_4 _(pH = 7.2-7.4), 1% polyvinilpirrolidone (PVP) and 0.02%, β-mercaptoethanol), to which 1% active carbon and 1% carborundum (600 mesh) were added, the latter as an abrasive.

When the fourth leaf was fully expanded, plants were inoculated on all expanded leaves with a cotton-tipped applicator, previously dipped in the inoculum. One negative control from each accession was inoculated only with inoculation buffer. To avoid escapes, one week later the plants were reinoculated on a new fully expanded leaf.

### Evaluation and virus detection

An apical leaf from each plant was collected at 15, 30, 45 and 60 days post inoculation (DPI). Leaf extracts were analysed by DAS-ELISA [58] using a specific antiserum of PVY (Loewe Biochemica GmbH, Sauerlach, Germany) to quantify the PVY-F14K virus. Viral accumulation was tested by the measurement of absorbance at 405 nm (A_405_) of the serologic reaction using a Model 550 Microplate Reader (Bio-Rad, Hercules, California, USA). Negative control extracts of each accession were used as negative samples for the ELISA test. A sample was considered positive for PVY (DAS-ELISA positive) when absorbance from the youngest leaf was three times higher than the absorbance mean of its negative control, and was classified as non-infected (DAS-ELISA negative) when the value of absorbance was below this threshold. The negative control mean was calculated for each accession with different time samplings (15, 30, 45 and 60 DPI). PVY symptoms on non-inoculated leaves were recorded visually and compared to the respective negative control at the same DPI. A symptom index was assigned to each plant according to the severity of the symptoms (leaf deformation, veinal chlorosis, mosaic, dark green vein-banding and stunted growth). This index ranged from 0 for plants with no symptoms to 4 for dead plants. An accession was considered resistant when no plants were infected systemically and no symptoms were observed, tolerant when showing DAS-ELISA positive in all or some plants but without symptoms and susceptible when DAS-ELISA positive with systematic symptoms. Once the survey ended, all material used in the screening (plants, pots and inoculum solution) were autoclaved at 121°C for 30 min to avoid viral contamination.

## Authors' contributions

VPI obtained the experimental data. JC designed the study and the experiments. VPI, FN and JC analysed the data and prepared the manuscript. All authors read and approved the final manuscript.

## Supplementary Material

Additional file 1 ***Capsicum *spp. accessions analysed by EcoTILLING and their haplotypes *eIF4E *and *eIF(iso)4E***. Accession number, country, species and haplotype variants of the *eIF4E *and *eIF(iso)4E *genes for all accessions analysed by EcoTILLING. Click here for file

Additional file 2 **Alignment of predicted eIF4E proteins from different haplotypes**. Alignment using Clustal X of predicted eIF4E proteins from different haplotypes detected by EcoTILLING and eIF4E protein of *pvr2^+ ^*allele [Genbank: AAM82190]. Click here for file

Additional file 3 **Alignment of predicted eIF(iso)4E proteins from different haplotypes**. Alignment using Clustal X of predicted eIF(iso)4E proteins from different haplotypes detected by EcoTILLING and eIF(iso)4E protein of *pvr6^+ ^*allele [Genbank: AAY62609]. Click here for file

Additional file 4 **Analysis to determine that translation initiation factor is involved in PVY resistance**. Response of the tested accessions against PVY-F14K according to their eIF4E and eIF(iso)4E proteins. Resistant accessions are highlighted in green, tolerant accessions in yellow and susceptible accessions in red. Click here for file

Additional file 5 **EcoTILLING gel image of first classification made to *eIF(iso)4E *gene**. Mutation detection of *eIF(iso)4E *gene in a gel image from IRD700 channel of LI-COR analyser, using CDP06433 sample as reference. Numbers on the left indicate the molecular weight marker in bp. (*) Self-hybridised reference sample. Click here for file

Additional file 6 **EcoTILLING gel image of second classification made to *eIF4E *gene**. Mutation detection of *eIF4E *gene in a gel image from IRD700 channel of LI-COR analyser, using CDP006188 sample of initial group 1 as reference. Numbers on the left indicate the molecular weight marker in bp. Numbers on the top indicate the EcoTILLING group after the first classification. (S) Self-hybridised reference sample. (*) Two accessions classified in group 2 or 3 after the first classification showing the same band pattern. (#) Two accessions classified in group 2 or 4 after the first classification showing the same band pattern. Click here for file
