# Supplementary material for: EcoTILLING in Capsicum species: searching for new virus resistances
Source: BMC Genomics. 2010 Nov 12;11:631. doi: 10.1186/1471-2164-11-631 (PMC3091766; doi:10.1186/1471-2164-11-631)
Supplement: Additional file 4 — Analysis to determine that translation initiation factor is involved in PVY resistance. Response of the tested accessions against PVY-F14K according to their eIF4E and eIF(iso)4E proteins. Resistant accessions are highlighted in green, tolerant accessions in yellow and susceptible accessions in red. [file 1471-2164-11-631-S4.PDF]

eIF4E protein	eIF(iso)4E protein A	B	C	D	E	F	G
A			CDP06188				
B		CDP01263	CDP09688<br>CDP04928	CDP09688<br>CDP01246			
C					CDP08791		
D		CDP02521					
E			CDP06433	CDP01135			
F	CDP00614	CDP05838	CDP06234<br>CDP00624	CDP00620			
G		CDP05436	CDP00973				
H		CDP08407					
I		CDP07700					
J							CDP00590
K	CDP04291		CDP02320				
L			CDP05581				
M			CDP06360			CDP07825	
N	CDP04865		CDP07490			CDP04131	
O	CDP09967		CDP09334				
P		CDP01186					
Q	CDP05929		CDP04710				
R		CDP06951					
