# Supplementary figures and images for: EcoTILLING in Capsicum species: searching for new virus resistances
Source: BMC Genomics. 2010 Nov 12;11:631. doi: 10.1186/1471-2164-11-631 (PMC3091766; doi:10.1186/1471-2164-11-631)

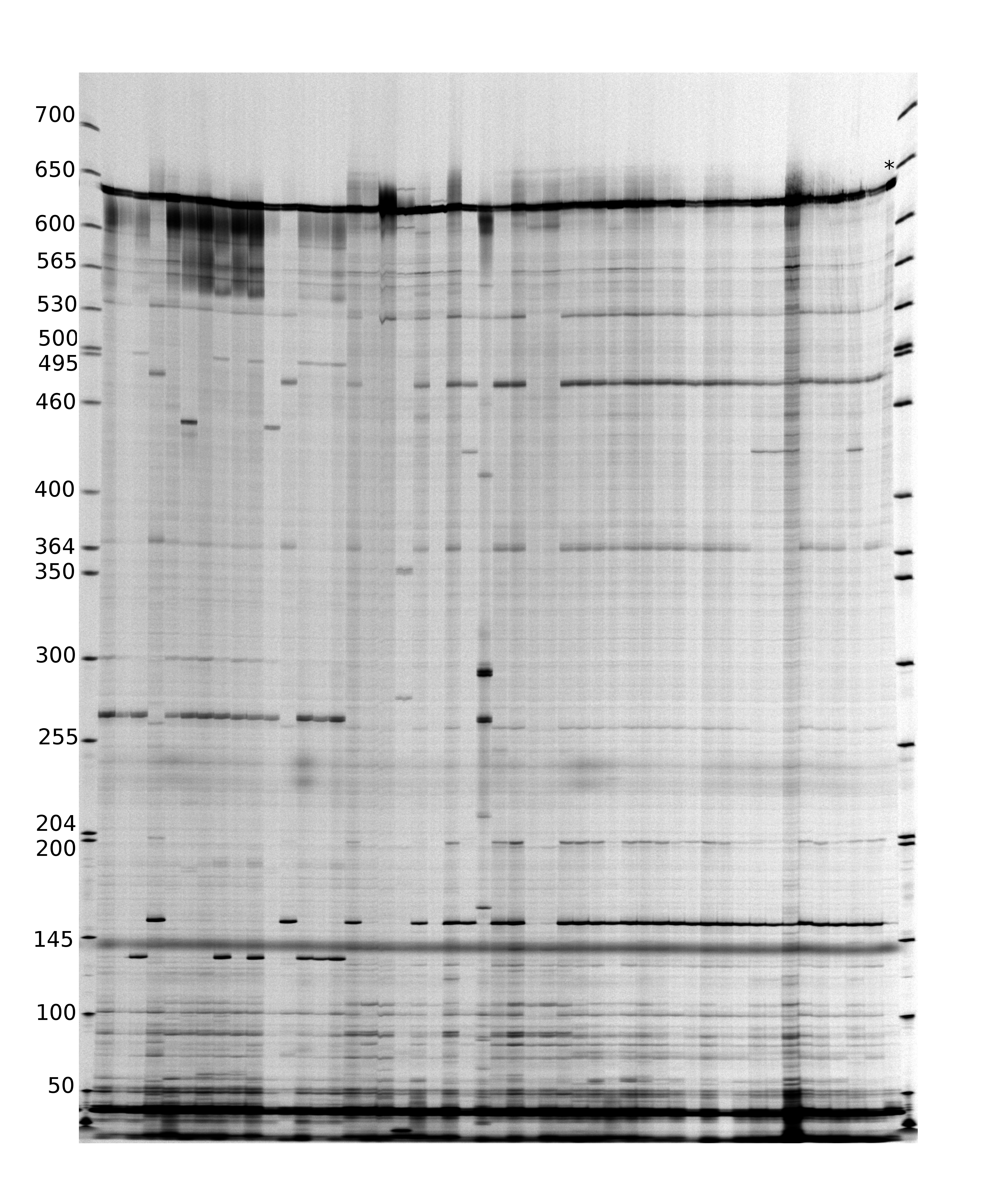

Supplement: Additional file 5 — EcoTILLING gel image of first classification made to eIF(iso)4E gene. Mutation detection of eIF(iso)4E gene in a gel image from IRD700 channel of LI-COR analyser, using CDP06433 sample as reference. Numbers on the left indicate the molecular weight marker in bp. (*) Self-hybridised reference sample. [file 1471-2164-11-631-S5.PNG]

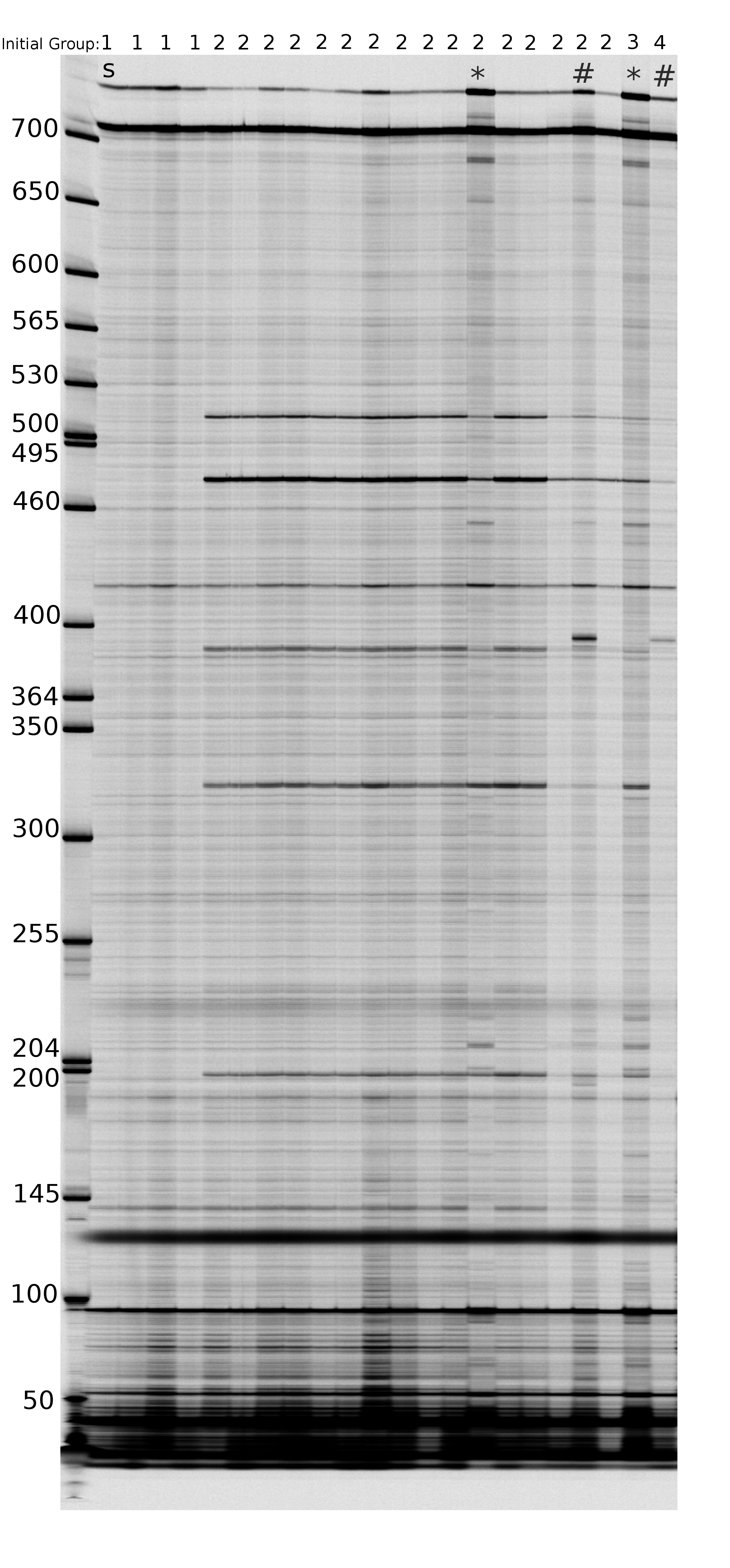

Supplement: Additional file 6 — EcoTILLING gel image of second classification made to eIF4E gene. Mutation detection of eIF4E gene in a gel image from IRD700 channel of LI-COR analyser, using CDP006188 sample of initial group 1 as reference. Numbers on the left indicate the molecular weight marker in bp. Numbers on the top indicate the EcoTILLING group after the first classification. (S) Self-hybridised reference sample. (*) Two accessions classified in group 2 or 3 after the first classification showing the same band pattern. (#) Two accessions classified in group 2 or 4 after the first classification showing the same band pattern. [file 1471-2164-11-631-S6.PNG]
